# Radiographic progression patterns and treatment response in adult H3 K27-altered diffuse midline glioma: a descriptive review and single center case series

**DOI:** 10.3389/fonc.2026.1934399

**Published:** 2026-10-26

**Authors:** Adam Lahrache, Hope M. Reecher, Aleksandra Winiarz, Savannah R. Duenweg, Daniel Kim, Allison K. Lowman, Biprojit Nath, Benjamin E. Chao, Fitzgerald Kyereme, Michael J. Barrett, Mrina Mtenga, Jennifer Connelly, Daniel Destiche, Mohit Agarwal, Elaine Tanhehco, Jamie Jacobsohn, Samuel A. Bobholz, Peter S. LaViolette

**Affiliations:** 1 Department of Radiology, Medical College of Wisconsin, Milwaukee, WI, United States; 2 Department of Biophysics, Medical College of Wisconsin, Milwaukee, WI, United States; 3 Department of Neurology, Medical College of Wisconsin, Milwaukee, WI, United States; 4 Department of Pathology, Medical College of Wisconsin, Milwaukee, WI, United States; 5 Department of Biomedical Engineering, Medical College of Wisconsin, Milwaukee, WI, United States

**Keywords:** ATRX, brain cancer, diffuse midline glioma, H3 K27, volumetric MRI

## Abstract

**Background:**

Diffuse midline glioma (DMG) with H3 K27 mutations are aggressive central nervous system tumors with limited treatment options and poor outcomes. Although more common in children, there is increasing recognition of adult cases, which remain under-characterized regarding longitudinal imaging trends and response to multimodal treatment. We aim to highlight heterogeneity and describe patterns of adult H3 K27-altered DMG in cases assessed at autopsy.

**Methods:**

Patients were included in the study if they were ≥ 21 years of age, and had a confirmed diagnosis of DMG, H3 K27-altered. Manual segmentations of contrast enhancement (CE) on T1-weighted post-contrast imaging and hyperintense FLAIR regions were performed at each timepoint. CE ratios (CE_vol_/FLAIR_vol_) were calculated to characterize enhancing versus infiltrative tumor burden. Treatment timelines were retrospectively aligned with imaging timing to evaluate disease progression and therapy response.

**Results:**

This case series included six patients with confirmed H3 K27-altered DMG. Longitudinal imaging data revealed radiographic heterogeneity between patients. Periods of FLAIR reduction were observed to occur around bevacizumab treatment. Mean CE ratios were lowest in Patients 5 and 1 (0.081 and 0.225 respectively), intermediate in Patient 4 (0.312), and highest in Patient 3 (0.668). ATRX-mutant cases (*n*, 2) had comparatively short overall survival of 26 and 164 days while ATRX-wild type cases (*n*, 4) survived 728-2,299 days.

**Conclusions:**

Among our cohort, we observed two patients with ATRX mutations to have comparatively short survival periods. Findings highlight heterogeneity of adult H3 K27-altered DMG, and limitations of imaging-based modalities to track tumor progression.

## 1 Introduction

Diffuse midline gliomas (DMG) are aggressive central nervous system tumors; those with H3 K27 mutations are classified as Grade 4 by the World Health Organization (WHO) regardless of characteristics on histology (1). The median overall survival (OS) for patients with this specific subtype of DMG is dismal, with survival estimates ranging between 10–14 months (2). These tumors often develop within the brainstem, thalamus, or spinal cord and progress with an infiltrative pattern which often limits surgical resection (3, 4). Poor blood-brain barrier penetration of treatments and inherent tumor resistance reduce treatment success (3–5).

Surgery, when feasible, and radiation therapy remain standard treatment, with a total dose of 54–60 gray (Gy) over six weeks providing limited benefit (4, 5). Few chemotherapy agents exist for use in cases of DMG. Temozolomide (TMZ) is an agent commonly used for glioblastoma patients, yet its benefit remains unclear in cases with unmethylated MGMT (5, 6). Lomustine (CCNU) crosses the blood-brain barrier and has utility for infiltrative disease (7). Bevacizumab, a vascular endothelial growth factor (VEGF) inhibitor, is used to reduce edema and improve symptoms in high-grade glioma (8, 9). Tumor Treating Fields (TTFields), a novel treatment, use alternating electric fields to disrupt mitosis and have improved survival and function in glioblastoma, yet data of their use in patients with DMG remains limited to case reports at this time (10, 11). Recent advancements have been made in the treatment of H3 K27-altered DMG, one such being the novel dopamine-receptor molecule inhibitor dordaviprone (ONC201), which shows promise in early adult reports (12).

The majority of published studies describe pediatric DMG, however the few adult cases of DMG have been reported to show distinct biology (3, 6, 13, 14). Patterns of progression and treatment response in adults are not well defined in comparison to other forms of brain cancer. To date, there is one robust systematic review comprised of 20 case reports and 135 unique adult patients with H3 K27-altered DMG (15). This review reports demographic and clinical characteristics such as presenting symptoms, genetic alterations, and survival analyses following treatment. Patients with an altered H3 K27 gene and ATRX mutations have been observed to have a more aggressive disease and worsened prognosis (13, 15). This case series reviews six adults with confirmed H3 K27-altered DMG diagnosis to report longitudinal magnetic resonance imaging (MRI)-based volume changes and treatment response on T1-weighted post-contrast (T1C) and Fluid Attenuating Inversion Recovery (FLAIR) sequences.

## 2 Methods

### 2.1 Study design and patient cohort

This study consisted of two components:

1. Clinical Case Review: Adult patients with confirmed H3 K27-altered DMG were identified from the Medical College of Wisconsin Neuro-Oncology Brain Bank. All confirmed cases were eligible for this component.
2. Imaging analysis: Patients eligible for the clinical case review portion of the study were subsequently screened for MRI scans suitable for volumetric analysis. To analyze volume over time, patients were required to have a minimum of three imaging timepoints available, and the lesion was required to be visible on both T1C and FLAIR images. Two patients were excluded: one patient who had fewer than three imaging timepoints due to a rapidly progressive clinical course, allowing for only one scan prior to passing, and one patient whose intracranial lesion had limited FLAIR or contrast-enhancing (CE) visibility as the bulk of the tumor was in the spinal cord throughout most imaging sessions prior to its metastasis to the brain, whereafter he passed shortly after and no subsequent images were obtained.

### 2.2 Chart review

Clinical data were collected by manual review of clinical reports extracted from each patient’s electronic medical record, including presenting symptoms, treatment history, radiographic progression, and autopsy or pathology findings. ATRX status was determined through Glioseq next-generation sequencing (16), which reported sequence mutations, if any, in this gene. Patients 5 and 6 had available histology with hematoxylin and eosin (H&E) stains, and immunohistochemistry stains probing for antigen Kiel 67 (Ki67), and (sex determining regionY)-box 2 (SOX2), demonstrating tumor cellularity and proliferation. Survival was calculated from the date of initial histologic diagnosis (i.e., following biopsy or resection for tissue) until death for overall survival, and until the first instance of radiographic recurrence for progression-free survival. Treatment events including surgery, radiation, chemotherapy (TMZ, CCNU), bevacizumab, and TTFields were extracted from clinical records and aligned with imaging timepoints to correlate radiographic and therapeutic changes.

### 2.3 Image analysis

All imaging was co-registered as part of standard processing for the institutional brain bank, with each patient’s serial studies registered to the final pre-mortem timepoint, providing a consistent spatial framework across imaging series. CE regions were segmented on T1C, and hyperintense regions on FLAIR images were segmented as well. All segmentations were performed manually in the axial plane using ITK-SNAP (version 4.0.0-alpha.3, 2022). Pre-contrast T1-weighted images were reviewed in tandem with post-contrast images at each timepoint to ensure intrinsically T1-hyperintense material was not misclassified as enhancement.

Initial segmentations were performed by a single author (ALa). All segmentations were reviewed by two neuroradiologists who inspected each timepoint against the source images and provided corrections where tumor boundaries were judged to be inaccurate. If disagreements occurred, the case was discussed until consensus was reached. All volumes reported are derived from consensus segmentations. Volumes were derived by multiplying the number of voxels in each mask by voxel dimensions and reported in mm³. The CE ratio was computed at each imaging timepoint by dividing CE volume by FLAIR volume. The value reported for each patient is the mean of these per-timepoint ratios. The CE ratio of CE_vol_/FLAIR_vol_ was chosen as an imaging metric by which active tumor burden could be represented and tumor heterogeneity could be quantified (17). Patients with lesions where the bulk is predominantly contrast-enhancing instead of FLAIR-hyperintense would have higher ratios, and those with greater FLAIR-hyperintensity (i.e., edema, inflammation) and less direct CE tumor burden would have lower ratios. As lesions are impacted by treatment such as surgery, radiation, or bevacizumab, the CE ratio in this study is to be understood as a limited imaging metric, rather than reflective of direct biological processes or cellular activity. Volumetric stability was defined as the longest interval during which the segmented volume remained within 10% of its value at the start of that interval, evaluated at each intervening timepoint. CE and FLAIR volumes were assessed independently. No minimum interval duration was enforced, as scan timing was determined by each patient’s clinical team as deemed medically necessary. Descriptive statistics were computed for CE, FLAIR, and CE ratio values for each patient.

## 3 Results

### 3.1 Clinical presentations and demographic features

Following screening, six patients with H3 K27-altered DMG were identified from the Neuro-Oncology Brain Bank, four of which were included in the imaging analysis portion (**Table 1**).

**Table 1:** Demographic and clinical information for the study cohort.

Patient	Sex	Age at death	Initial tumor location	Final pathologic diagnosis	Molecular alterations	Treatment history	PFS (days)	Overall survival (days)
1	F	27	Left thalamus	DMG, G 4	H3 K27– altered, IDH wt	Bevacizumab, TMZ, XRT, PLDR	1,589	2,299
2	M	38	Spinal cord: lower thoracic to upper lumbar region	DMG, G 4	H3 K27–altered	Bevacizumab, TMZ, XRT to thoracic and lumbar spine	1,139	1,802
3	M	53	Right thalamus	DMG, G 4	H3 K27–altered IDH1 wt	Bevacizumab, TMZ, XRT, TTF, CCNU	1,234	1,695
4	M	42	Right thalamus	DMG, G 4	H3 K27–altered, ATRX-mutant	Bevacizumab	68	164
5	M	57	Right temporal, left basal ganglia, thalamus	DMG, G 4	H3 K27–altered, TP53mutant	TMZ, IMRT	191	728
6	M	38	Left pons	DMG, G 4	H3 K27–altered, ATRX-mutant	IMRT	NA*	26

Key: PFS, progression-free survival; DMG G 4, diffuse midline glioma; WHO Grade 4, XRT, conventional external beam radiation; TMZ, temozolomide; PDLR, pulsed low dose radiation, CCNU, lomustine; IMRT, intensity-modulated radiation therapy.

*Patient passed away prior to radiographic recurrence.

#### 3.1.1 Patient 1

A 27-year-old female was diagnosed with a left thalamic-infiltrating WHO Grade 4 astrocytoma that was H3 K27-altered per 2007 classification (18). Her initial treatment included radiation and TMZ. Subsequent imaging showed radiographic progression 1,589 days after initial diagnosis. She then developed decreased mobility and cognition and entered hospice 532 days after progression was identified. She died 2,299 days after diagnosis. Autopsy showed viable tumor in the left thalamus, right hypothalamic area, and deep white matter. Neuronal loss and necrosis in the corpus callosum, pons, bilateral thalamus, and hippocampus were interpreted as treatment effect.

#### 3.1.2 Patient 2

A 38-year-old male presented with morning nausea, diplopia, loss of left-sided sensation below L2, and no muscle contraction in the left lower extremity. Imaging revealed a lesion spanning levels T11–L1 of the spinal cord. He underwent laminectomy and biopsy, for which pathology showed WHO Grade 2 diffuse astrocytoma, IDH-1 wild type on immunohistochemistry. He completed radiation with TMZ. Progression of the tumor occurred on radiographic imaging 1,139 days after initial diagnosis. He remained asymptomatic and deferred surgery yet developed leg weakness three months later and underwent a second debulking 1,267 days after the first surgery. Genetic testing completed at an outside hospital showed H3 K27-altered and BRAF-negative. After surgery, he developed new right leg weakness and worsening left leg weakness. Three months later, imaging showed a second instance of progression, and TMZ with bevacizumab was started. One year later, he developed dizziness, headaches, and seizures, and was referred to hospice. Overall survival was 1,802 days. Autopsy revealed viable tumor in the cerebellum, hippocampus, temporal lobe, and around the pineal gland.

#### 3.1.3 Patient 3

A 53-year-old male presented with gradual-onset left-sided weakness, impaired balance, and slurred speech. MRI showed a 4.5 cm heterogeneously enhancing right thalamic mass. Biopsy diagnosed WHO Grade 4 glioblastoma per updated classification (1). He received radiation and TMZ, then CCNU, bevacizumab, and TTFields six months later. GlioSeq molecular analysis identified IDH wild-type, H3F3A, TP53, and NF1 mutations, confirming H3 K27-altered DMG. After 1,234 days from initial diagnosis, imaging showed progression with a new cerebellar lesion. He developed cerebellar ataxia, worsening confusion, and new left-sided weakness, subsequently entering hospice 428 days after radiographic recurrence. He continued to decline with visual hallucinations, excessive sleep, and loss of responsiveness. Overall survival was 1,695 days. Autopsy revealed viable tumor in the right thalamus, extending to the right putamen, corpus callosum, midbrain, pons, cerebellar vermis, and left cerebellar hemisphere.

#### 3.1.4 Patient 4

A 42-year-old male presented with memory loss, attention problems, and slowed cognitive processing. MRI revealed an enhancing right thalamic lesion. He then underwent surgical resection and biopsy, from which tissue was sent for GlioSeq molecular testing, which identified H3F3A, FGFR1, and ATRX mutations, CDK4, MYCN, and PTEN copy number alterations, and copy-neutral loss of heterozygosity at 17q and 19q. This constellation of molecular findings was reported as consistent with H3 K27-altered DMG. One month after resection, he underwent a second resection, cyst fenestration, and ventriculoperitoneal shunt placement. Disease progression followed 68 days after initial diagnosis. The patient chose symptom management only and received bevacizumab. His overall survival was 164 days. Autopsy showed viable tumor in the right thalamus, frontal white matter, basal ganglia, putamen, midbrain, corpus callosum, pons, cerebellar vermis, and left cerebellar hemisphere, with separate nodules in the cerebellum and medulla.

#### 3.1.5 Patient 5

A 56-year-old male presented with intermittent double vision and cognitive decline. MRI revealed multifocal non-enhancing FLAIR abnormalities in the right temporal lobe, left basal ganglia, and thalamus. He underwent biopsy for pathology tissue analysis with GlioSeq, which identified TP53 and H3F3A mutations, RB1 and SMARCA4 copy number loss, and copy-neutral loss of heterozygosity at 16q, consistent with H3 K27-altered DMG. He received intensity-modulated radiation therapy, adjuvant TMZ, and CCNU. Bevacizumab was given for three months, paused for six months after an acute stroke, then restarted for seven months, held for three months, and given once more. He passed away 728 days after diagnosis. Autopsy revealed viable tumor in the left temporal lobe, thalamus, hippocampus, midbrain, left cerebellum, right frontal lobe, right insula, and anterior left pons (**Figure 1**). Radiation atypia and necrosis were present in the bilateral frontal lobes, right insula, and anterior left pons.

**Figure 1:**
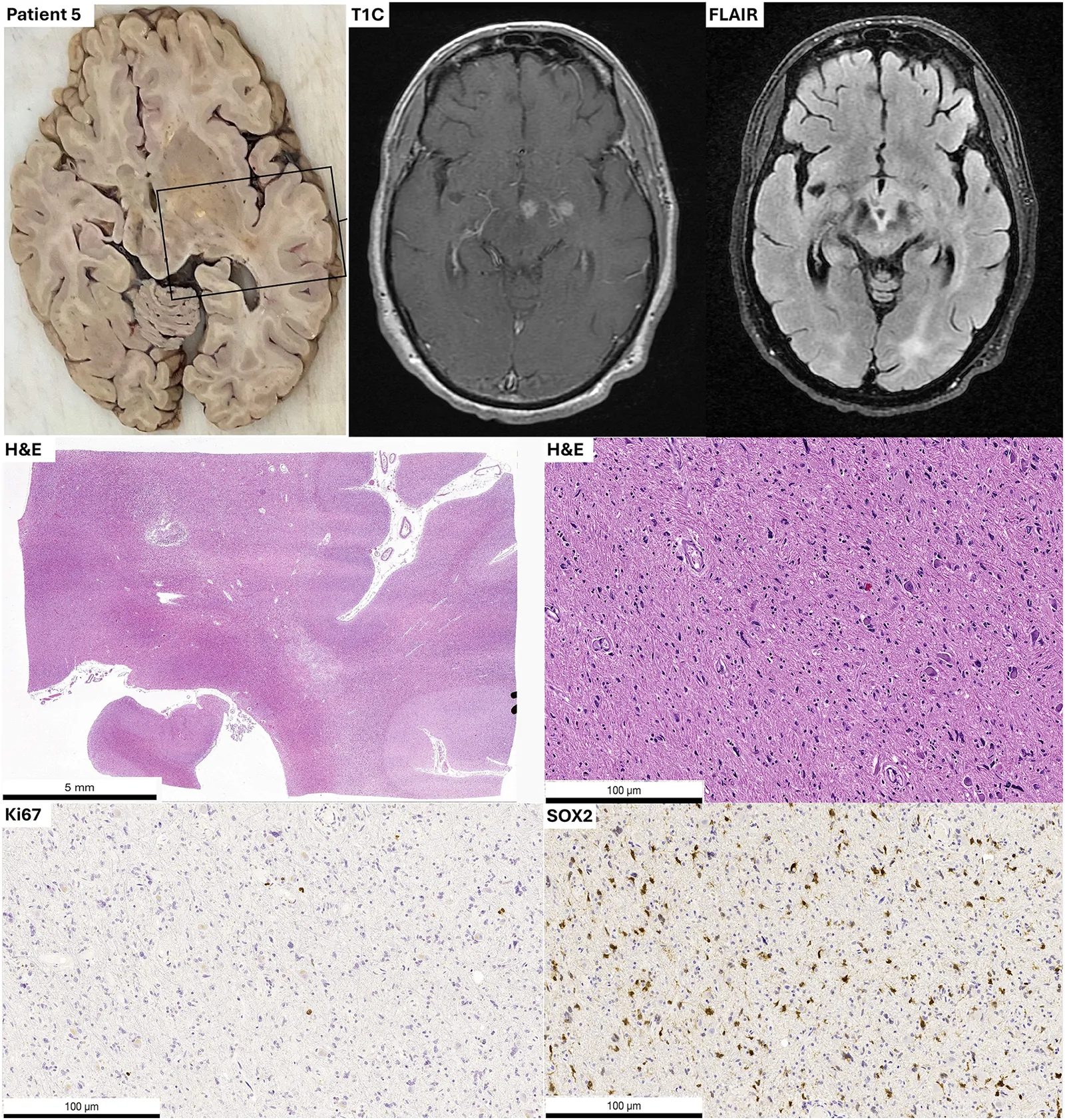
Histology from patient 5 autopsy with H&E, SOX2, and Ki67 staining. Histology from Patient 5, sample taken from the left thalamic area with extensive SOX2 and Ki67 positivity seen, indicating high levels of stem cell pluripotency and cell proliferation in the area of observable tumor on the patient’s imaging.

#### 3.1.6 Patient 6

A 38-year-old male presented with intermittent horizontal diplopia. Imaging revealed a left pontine lesion. Biopsy was completed, with tissue sent for GlioSeq molecular analysis which showed H3F3A, NF1, PIK3CA, and ATRX mutations with 2p/2q/7p/7q/17q gains and 8p loss, consistent with H3 K27-altered DMG. Radiation therapy was initiated 17 days after surgery. Six days later, the patient presented in rapid clinical decline of severe physical weakness, inability to perform daily activities, loss of ambulation, difficulty swallowing, severe dysarthria, deconjugate gaze, bilateral facial weakness, and global motor weakness. Hospice care was initiated 23 days after initial diagnosis. He passed away three days later, 26 days after diagnosis. Autopsy revealed viable tumor in the left cerebellar peduncle, midbrain, pons, medulla, and basal ganglia (**Figure 2**).

**Figure 2:**
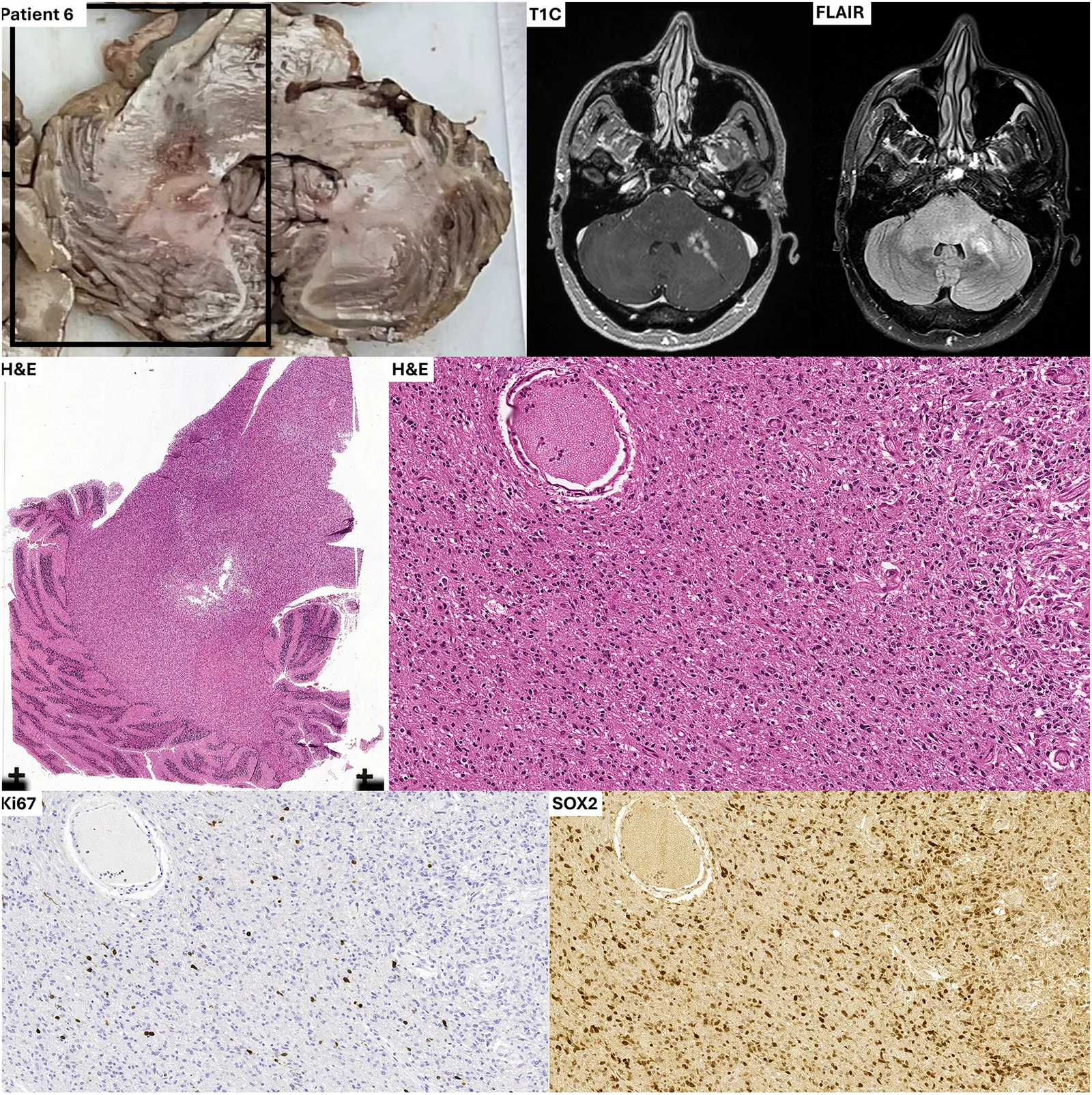
Histology from patient 6 autopsy with H&E, SOX2, and Ki67 staining. Histology from Patient 6, sample taken from the right cerebellar region. Extensive SOX2 and Ki67 positivity indicates high levels of stem cell pluripotency and cell proliferation in the area of observable tumor on the patient’s imaging. H&E shows extensive cellularity.

### 3.2 Survival: ATRX mutations and progression-free survival

The median overall survival for the cohort was 1,211.5 days (range: 26 to 2,299 days). Two patients harbored ATRX mutations and had overall survival periods of 26 and 164 days. The remaining four patients were ATRX-wild type and had overall survival ranging from 728 days to 2,299 days. The ATRX-wild type group also comprised the multimodal therapy group (*n*, 4), receiving combinations of radiation, chemotherapy, bevacizumab, or TTFields. ATRX-mutant patients received monotherapy.

Three patients, all being ATRX-wild type, experienced extended periods of progression-free survival based on radiographic and clinical data: 1,589 days of progression free survival in Patient 1, 1,139 days in Patient 2, and 1,234 days Patient 3.

### 3.3 Imaging and volumetric results

#### 3.3.1 Patient 1

Across 22 MRI scans over 1,720 days, mean FLAIR volume was 4,872 mm³ and CE volume 1,401 mm³ (ratio 0.225). Radiographic stability persisted for 139 days in FLAIR and 98 days in CE. FLAIR and CE volumes decreased early before a major increase at day 1,190, with final volumes reaching 20,357 mm³ and 4,088 mm³, respectively (**Figure 3A**).

**Figure 3:**
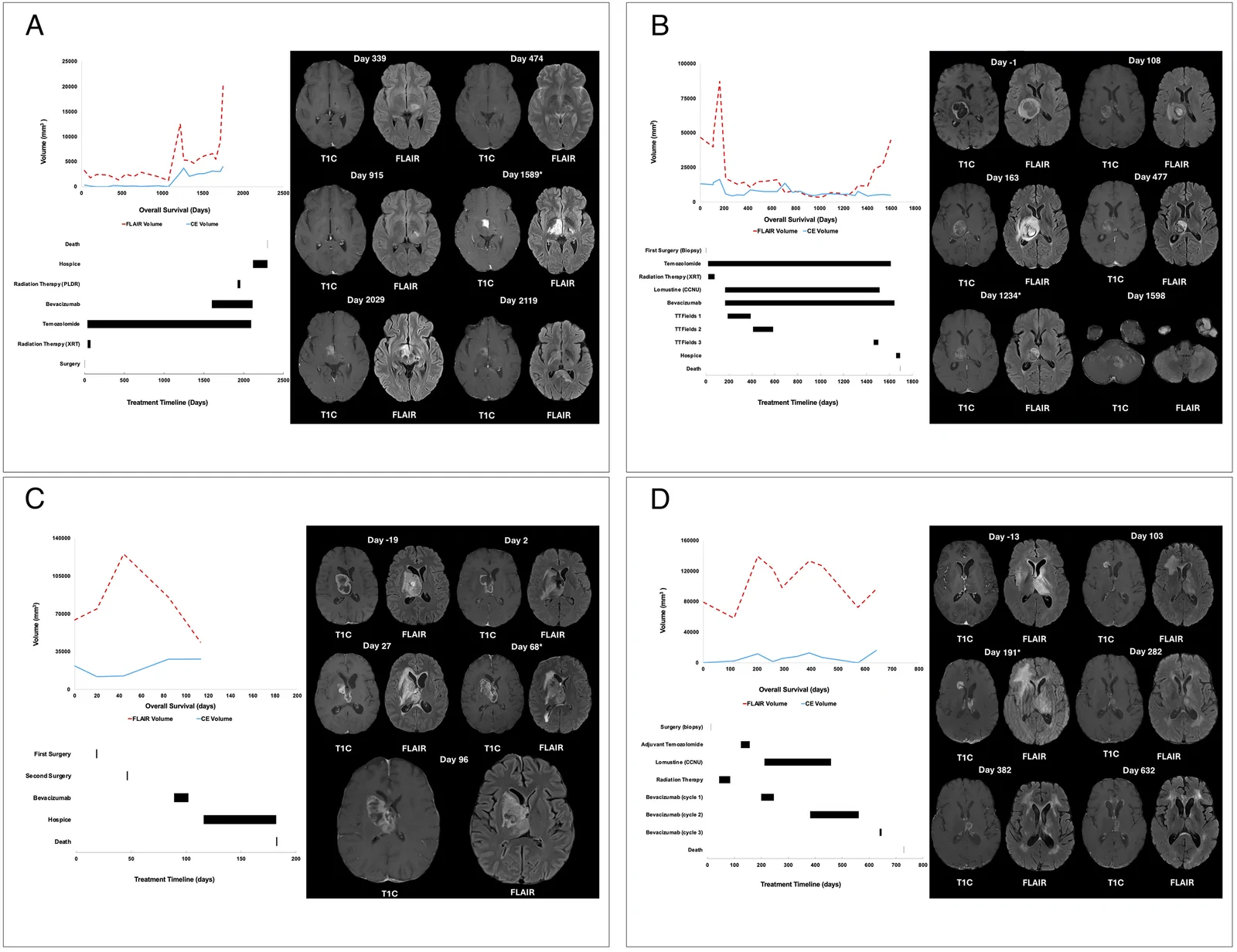
**(A–D)** Longitudinal imaging, volumetric trends, and treatment course of four adult patients with H3 K27-altered DMG. *Dates above scan images are reflective of the number of days since confirmed diagnosis. The asterisk denotes the scan at which radiographic progression ocurred. **(A)** Patient 1 - Patient exhibited minimal CE and hyperintensity until progression was observed on imaging obtained 1,598 days, after initial diagnosis, with a new hyperintense and enhancing lesion. An increase in FLAIR hyperintensity burden was observed at the last imaging timepoint. Treatment timeline annotated with all major interventions shows the patient received radiation therapy and TMZ early, bevacizumab and additional radiation therapy later on before transitioning into hospice care. Imaging shows minimally enhancing tumor early on with new avidly enhancing lesion seen on day 1,598 and beyond. **(B)** Patient 3 - FLAIR signal initially rose sharply before stabilizing, while CE volume remained suppressed following initial treatment, with minimal change throughout follow-up. The patient’s treatment course included surgery, temozolomide, lomustine, bevacizumab, tumor treating fields, and eventually transitioned to hospice. Sustained CE suppression with fluctuating FLAIR suggests infiltrative progression with minimal enhancement. New enhancing cerebellar lesion was seen on imaging day 1,234, which was reflective of radiographic progression. This lesion is best visualized on the scan obtained at day 1,598. **(C)** Patient 4 - Normalized FLAIR and CE volumes show early increase in FLAIR with declining CE volume post-operatively, followed by rapid CE rebound and FLAIR reduction after reoperation and bevacizumab initiation. Treatment course included two surgeries, short-term bevacizumab, and hospice care. CE rebound despite FLAIR decrease may suggest enhancement recurrence with treatment resistance. Imaging shows before and after patient underwent two resections (initial resection on day 18, subsequent resection with debulking on day 46). Radiographic progression was seen on the scan obtained 68 days after diagnosis. **(D)** Patient 5 - Normalized FLAIR and CE volumes show initial increase in both compartments. CE volume dropped sharply post-bevacizumab (day 199) before rebounding, while FLAIR progressed gradually and plateaued. The patient underwent surgery, adjuvant chemotherapy (temozolomide and lomustine), radiation, and three cycles of bevacizumab. The radiographic trajectory highlights non-linear treatment response and a transition toward infiltrative behavior over time, with progression 191 days after diagnosis. *Note: Dates above scan images are reflective of the number of days since confirmed diagnosis. The asterisk denotes the scan at which radiographic progression occurred.

#### 3.3.2 Patient 3

Across 27 MRI scans over 1,598 days, mean FLAIR volume was 19,150 mm³ and CE volume 7,528 mm³ (ratio 0.668). Radiographic stability was observed for 168 in FLAIR and 218 days in CE. Volumes rose initially, then declined markedly before stabilizing. Final radiographic tumor volumes were 45,000 mm³ for FLAIR, and 4,920 mm³ for CE (**Figure 3B**).

#### 3.3.3 Patient 4

Across 5 MRI scans over 113 days, mean FLAIR volume was 78,840 mm³ and CE volume 20,390 mm³ (ratio 0.31). FLAIR increased early while CE decreased, followed by a reversal of this pattern at the final MRI (**Figure 3C**).

#### 3.3.4 Patient 5

Across 10 MRI scans over 643 days, mean FLAIR volume was 103,030 mm³ and CE volume 8,330 mm³ (ratio 0.081). Both volumes fluctuated substantially, showing alternating increases and decreases across timepoints. Final FLAIR and CE volumes were 84,780 mm³ and 24,500 mm³ (**Figure 3D**).

## 4 Discussion

### 4.1 Patient clinical courses and outcomes

In this cohort of six adults with H3 K27-altered DMG, the two ATRX-mutant patients were observed to have comparatively short overall survival (26 and 164 days), while ATRX-wild type cases survived 728–2,299 days. These findings align with two prior studies including a robust systematic review, which identified ATRX mutation as a negative prognostic factor (13, 15). Given the small sample and limited existing data, these results must be interpreted cautiously and validated in larger studies.

Treatment intensity also varied across this small cohort. All four ATRX-wild type patients received multimodal therapy combining radiation, chemotherapy, bevacizumab, or TTFields, while both ATRX-mutant patients received a single treatment. This pattern may suggest treatment intensity reflected disease course, rather than acting as the primary driver of survival differences. As there again are only two patients in the ATRX-mutant cohort, no prognostic conclusions can be drawn from this study, rather descriptive observations only. Extended progression-free intervals were observed only in ATRX-wild type patients, although tumor location and comorbidities may also have contributed. These findings support the need for larger, molecularly stratified studies to determine whether ATRX mutation independently predicts prognosis.

Bevacizumab is known to complicate imaging assessment for tumor response (19). Bevacizumab will decrease areas of contrast enhancement, due to decreased vasculature or blood flow that enhances, which may appear similar to decreased contrast enhancement reflective of true tumor cell death (20). There is likely a combination of these processes concomitantly occurring during periods of decreased contrast enhancement whilst on bevacizumab. Such effects from anti- VEGF therapy, like bevacizumab, may impact CE volumes as assessed in our study. Due to lack of cellular information unobtainable from traditional MRI scans, firm conclusions cannot be made regarding what increased or decreased areas or amounts of contrast enhancement reflect, particularly in study cases with patients receiving anti-VEGF therapy. Decreases in CE volume, subsequently decreasing the CE ratio itself, may therefore reflect lessened blood flow within the tumor microvasculature rather than directly reflecting tumor cell death and decreased CE volume from cell necrosis. However, there would likely be some interplay of these, as decreased blood flow from anti-VEGF therapy would inevitably cause ischemia, leading to cell death which similarly would decrease the CE volume.

### 4.2 Volumetric analysis

This case series highlights the heterogeneity in radiographic progression and treatment response among adults with H3 K27-altered DMG. Bevacizumab use was associated with reduction in FLAIR volume in four patients, most notably in Patient 4 (day 44 to end of treatment) and Patient 5 (day 202 to 258) (**Figures 3C, D**). CE volumes did not consistently decline and was occasionally observed to rebound, which may reflect this tumor subtype’s heterogeneity in true progression or treatment response. The mean CE ratio was lowest in Patient 5 (0.081), followed by Patient 1 (0.225) and Patient 4 (0.312), and highest in Patient 3 (0.668), indicating a greater proportion of non-enhancing abnormality in lower ratio cases.

Volumetric stability also varied: Patient 3 experienced stability for 168 days (FLAIR) and 218 days (CE), Patient 1 for 139 days in FLAIR and 98 days in CE, Patient 4 for 29 days in CE only, and Patient 5 for 47 days in FLAIR only. These differences reflect individualized treatment courses and emphasize the value of tracking both CE and FLAIR when assessing progression in H3 K27-altered DMG.

This study did not utilize the RANO 2.0 or earlier RANO-HGG criteria as strict criteria by which treatment response was assessed, as the primary function of this study was to report general longitudinal imaging trends of this small adult cohort, not to assess treatment response of DMG to a varied array of treatments represented herein. Radiographic progression was determined by attending radiologists on scan reports and conferred by the patient’s primary neuro-oncologist upon clinical imaging review and was not sub-categorized into complete or partial response. Our small sample size negates any meaningful conclusions that may attempt to be drawn from retrospectively assessing volumetric changes against RANO criteria. Future studies should include larger sample sizes and comparison to established response criteria, such as RANO-HGG or 2.0 (21, 22). In addition, future studies should be prospective in nature, multi-institutional, and collect volumetric imaging paired with molecular information from tissue so that the CE ratio may be validated as a potential biomarker for tumor response.

### 4.3 Limitations

Interpretation of these findings is limited by sample size and methodology. The small cohort of six adult patients restricts generalizability and impacts statistical analysis. In addition, the six adult patients included in this study represent tumors of varied regions (i.e., thalamus, pons, spine), and, due to the nature of high-grade tumor treatment regimens, had varied treatment protocols. Due to this, it may be difficult to generalize findings of this case series to the larger group of adult H3 K27-altered DMG. Segmentations were manual, and reproducibility was not assessed. Each timepoint was segmented once prior to review rather than independently by multiple readers, so neither intra- nor inter-observer agreement could be computed for this series. Expert neuroradiologist reviews and corrections of segmentations mitigate but do not eliminate all possible errors. Patient selection bias may be present, as some were excluded for limited imaging or lack of measurable tumor signal. Finally, FLAIR and CE changes are merely surrogate markers of disease activity and may not fully reflect underlying tumor biology or treatment effects, such as increased FLAIR following acute stroke in Patient 5, or postoperative edema following cyst fenestration in Patient 4.

## 5 Conclusion

In this case series, comprised of six adult H3 K27-altered DMG patients, longitudinal MRI using CE and FLAIR segmentation with CE ratios was utilized as a quantitative metric to evaluate enhancing tumor from peritumoral hyperintensity and allowed another form of observing clinical courses of patients. We observed transient FLAIR reduction during periods of bevacizumab treatment, and such FLAIR changes occasionally occurred without observing concurrent change in CE regions. Importantly, given the small sample, causal links cannot be drawn regarding ATRX status or tumor ratios. Larger, ATRX-stratified studies using standardized volumetric methods are needed to confirm these patterns and test their relationship to survival outcomes.

## Data Availability

The raw data supporting the conclusions of this article will be made available by the authors, without undue reservation.
